# Editorial: Cerebral autoregulation and neurovascular coupling in brain disorders

**DOI:** 10.3389/fneur.2022.1001342

**Published:** 2022-09-09

**Authors:** Xiuyun Liu, Marek Czosnyka, Chiara Robba, Dong Ming

**Affiliations:** ^1^Academy of Medical Engineering and Translational Medicine, Tianjin University, Tianjin, China; ^2^Department of Biomedical Engineering, College of Precision Instruments and Optoelectronics Engineering, Tianjin University, Tianjin, China; ^3^Department of Clinical Neurosciences, University of Cambridge, Cambridge, United Kingdom; ^4^Institute of Electronic Systems, Warsaw University of Technology, Warsaw, Poland; ^5^Department of Surgical Sciences and Integrated Diagnostics (DISC), University of Genoa, Genoa, Italy; ^6^San Martino Policlinico Hospital, Istituto di Ricovero e Cura a Carattere Scientifico for Oncology and Neuroscience, Genoa, Italy

**Keywords:** cerebral autoregulation, neurovascular coupling, multi-modality monitoring, neuro intensive care unit, brain disorders

## Introduction

Although the brain represents only 2% of our body weight, it consumes 20–25% of the total oxygen and nutrition of the body (1). Keeping adequate and constant blood supply to the brain is extremely important. This ensures the stable delivery of oxygen and other substances and the removal of the waste products of brain metabolism, especially in patients with brain disorders. Two important mechanisms involved in cerebral hemodynamic management are cerebral autoregulation (CA) and neurovascular coupling (NVC). Cerebral autoregulation refers to the ability of the brain to keep stable cerebral blood flow despite changes in cerebral perfusion pressure or arterial blood pressure (2), while NVC adapts cerebral blood flow in accordance with local neuronal activities [Liu et al.; (3)]. These two functions may be disturbed in patients with brain disorders in neuro intensive care units (NICU). The brain disorders could include stroke, hypoxia, traumatic brain injury (TBI), or subarachnoid hemorrhage (SAH). The damaged cerebral arterioles and capillaries in these patients would result in a lack of blood supply to the brain which may further aggravate cell death *via* blood-brain-barrier damage, inflammatory response, and endothelial cell and receptor dysfunction.

The early detection of the functional impairment of CA and NVC would enable early-stage decisions and prevent brain deterioration such as secondary brain injury, vasospasm, delayed cerebral infarction (DCI) after SAH, and sudden and severe headaches due to vessel constriction in reversible cerebral vasoconstriction syndrome (RCVS). Therefore, effective and prompt monitoring of brain blood perfusion and oxygenation with the quantitative metrics of CA or NVC assessment is in urgent need. Moreover, the mechanisms of impaired CA and NVC in brain disorders, the influence of clinical interventions on these two functions, and the effect of personalized treatment based on CA or NVC still need further investigation.

This Research Topic aims to provide the most recent update on the basic science of, and advanced techniques for, CA and NVC. The issue currently includes 24 papers on the dynamic mechanism of CA, new techniques for CA and NVC monitoring, the cellular mechanism of NVC, and reviews or commentaries on the current progress in this field. The papers cover several fields, including physics, biomedical engineering, clinical engineering, critical care medicine, pathology, and translational studies.

## Novel techniques for CA monitoring

This thematic section introduces various novel and updated techniques for quantitative CA assessment, including transcranial doppler (TCD), near-infrared spectroscopy (NIRS), electroencephalograph (EEG), retina vessel diameter, and magnetic resonance imaging (MRI), aiming to find a non-invasive, efficient, and appropriate method for CA assessment applied at a patient's bedside.

Warner et al. provide a detailed temporal analysis of impaired cerebral vascular reactivity to hypercapnia after SAH in rats. They highlight the promising role of retinal vessel diameter as a non-invasive screening tool after SAH. Eleveld et al. investigate a newly developed NIRS-based CA index, i.e., oxygenated–deoxygenated hemoglobin phase differences in the (very) low frequency (VLF/LF) in an endotoxemia study population. The results show a reversible decrease in NIRS-derived CA phase difference after endotoxin infusion, suggesting that endotoxin administration in healthy participants reversibly impairs CA, accompanied by sustained microvascular vasodilation. Pham et al. applies a hemodynamic model of coherent hemodynamics spectroscopy (CHS) to assess CA in five healthy subjects and 3 NCCU patients. They demonstrate the feasibility of measuring coherent de-oxygenated hemoglobin concentrations and blood pressure oscillations to assess autoregulation in NICU patients. However, in a study by Robba, Cardim et al., they report that changes in arterial-oxyhemoglobin components detected by NIRS had the highest accuracy to assess CBF changes. Nonetheless, these studies propose the use of indexes derived from the different components of rSO_2_ for the bedside evaluation of cerebral hemodynamics.

## CA in different brain disorders with clinical interventions

In this section, the authors investigate the changes in CA in patients with different brain disorders, such as acute ischemic stroke, aneurysmal subarachnoid hemorrhage (aSAH), steno-occlusive disease, and even COVID-19. They aim to investigate the influences of brain disorders on cerebral vasculature and cerebral perfusion to better understand the pathogenesis.

Uryga et al. investigate the influence of aSAH on the relationship between baroreflex sensitivity (BRS) and CA, which normally shows a compensatory interaction in healthy volunteers. However, this inverse relationship was lost in patients who developed cerebral vasospasm after aSAH, both before and during vasospasm.

Wu et al. report reduced network degree, local and global efficiency, and enhanced modularity in the contralateral normal hemispheres of steno-occlusive disease patients, compared with healthy volunteers using resting-state blood oxygen level-dependent (BOLD) imaging.

COVID-19 became a pandemic in 2020 and studies have shown the long-term impact of COVID-19 on the lungs, heart, and cognition. In this special collection, Robba, Messina et al. test the effects of the passive leg-raising test, fluid challenge, and norepinephrine on CA and oxygenation in critically ill COVID-19 patients. The study showed that the PLA test introduced adverse effects in CA in COVID-19 patients on a ventilator.

Concerning the influences of other treatments on CA, Wellard et al. investigate the changes of volume pressure compensation indices, cerebrovascular pressure reactivity indices, and heart rate variability indices after hyperosmolar admission in 30 children. They conclude that bolus therapy of hyperosmolar without preceding intracranial hypertension may alter cerebral dynamics by increasing intracranial pressure and decreasing pressure-volume compensation.

Yao et al. report a negative relationship between successive blood pressure variations and 3-month neurological outcome in acute ischemic stroke patients with intracranial artery stenosis or occlusion (SIASO) treated with intravenous thrombolysis. However, this relationship does not exist in patients without SIASO.

## Novel techniques for NVC monitoring

This thematic section focuses on the new techniques for NVC assessment *via* either animal models or human studies. Albanna et al. introduce a new method to non-invasively assess NVC *via* retinal vessel analysis in patients after aSAH. They report that aSAH results in sustained impairments of NVC in the retina and characteristic changes in the kinetics of retinal arterial responses may be associated with delayed cerebral ischemia. Meanwhile, based on similar techniques, the same research group (Neumaier et al.) compare the response of retinal vessels during flicker stimulation between Cav2.3-deficient mice and control mice. They propose that Cav2.3 channels could be involved in NVC and may contribute to the impairment of vasomotor responses under pathophysiological conditions.

Seker et al. present a fast, reliable, and unbiased data processing tool for the analysis of NVC. This was assessed by laser speckle contrast imaging and two-photon microscopy. Using the new analysis tool, they find that NVC is differently affected during the aging process in mice, with maximal NVC reached in 1-year-old mice compared with mice aged 6–8 weeks and 2 years old.

## Review articles in CA and NVC

This special collection also includes several review articles on the most up-to-date information about CA and NVC.

Longhitano et al. systematically review CA in three groups of non-brain injured patients: sepsis and septic shock, during surgery, and in pediatric population. They summarize that impaired CA may result in cognitive dysfunction, neurological damage, worse outcomes, and increased mortality. The authors conclude that monitoring CA might be a useful tool for the optimization of bedside treatment and the individualization of the clinical management of this group of patients.

Spilka et al. present a perspective on CA monitoring in neonatal cardiac surgery requiring cardiopulmonary bypass. They review the technical considerations for CA monitoring in the operating room and point out two requirements for cardiac surgery: that it should be hands-free and non-invasive, which reflects the advantage of using NIRS for CA monitoring.

Szczygielski et al. review the role of the Aquaporin-4 as a gatekeeper, regulating the water exchange between intracellular space, glymphatic system, and intravascular compartment. They point out that AQP4 as the key component of cerebral fluid homeostasis, acting not only as a passive channel for water and small molecular substances but playing a key role in the proper functioning of the blood-brain barrier and perivascular unit. As such, adapting the glymphatic flow to the phases of neuronal activity with increased blood flow demand is shown to be important.

## Conclusion

In conclusion, this special collection provides the most recent findings about CA and NVC, focusing on new assessment techniques, basic mechanisms of CA and NVC functions, comparisons of CA or NVC between patients with brain diseases and healthy controls, and the change in CA and NVC during neuroprotective interventions. The collection highlights the importance of CA and NVC monitoring in NICU, which could potentially enable early treatment and improve patient outcomes. Nevertheless, great challenges in this field exist and the following are needed: (1) a standardized method for CA and NVC assessment at the patient's bedside; (2) multi-disciplinary knowledge to better understand the pathologies and course of cerebral vasculature changes over time in different brain diseases; (3) clinical trial validation for these novel techniques for patient-personalized treatment to improve patients' clinical outcomes.

## Author contributions

All authors listed have made a substantial, direct, and intellectual contribution to the work and approved it for publication.

## Funding

This study was supported by the National Science and Technology Major Project of the Ministry of Science and Technology of China (No. 2021YFF1200600) and the Tianjin University Foundation (No. 0903069003 and No. 0903069004).

## Conflict of interest

The authors declare that the research was conducted in the absence of any commercial or financial relationships that could be construed as a potential conflict of interest.

## Publisher's note

All claims expressed in this article are solely those of the authors and do not necessarily represent those of their affiliated organizations, or those of the publisher, the editors and the reviewers. Any product that may be evaluated in this article, or claim that may be made by its manufacturer, is not guaranteed or endorsed by the publisher.
